# Supplementary material for: Collagen-rich omentum is a premetastatic niche for integrin α2-mediated peritoneal metastasis
Source: eLife. 2020 Oct 7;9:e59442. doi: 10.7554/eLife.59442 (PMC7541088; doi:10.7554/eLife.59442)
Supplement: Transparent reporting form [file elife-59442-transrepform.docx]

***eLife’s* transparent reporting form**

We encourage authors to provide detailed information *within their submission* to facilitate the interpretation and replication of experiments. Authors can upload supporting documentation to indicate the use of appropriate reporting guidelines for health-related research (see [EQUATOR Network](http://www.equator-network.org/%20)), life science research (see the [BioSharing Information Resource](https://biosharing.org/)), or the [ARRIVE guidelines](http://www.plosbiology.org/article/info:doi/10.1371/journal.pbio.1000412) for reporting work involving animal research. Where applicable, authors should refer to any relevant reporting standards documents in this form.

If you have any questions, please consult our Journal Policies and/or contact us: [editorial@elifesciences.org](mailto:editorial@elifesciences.org).

**Sample-size estimation**

- You should state whether an appropriate sample size was computed when the study was being designed
- You should state the statistical method of sample size computation and any required assumptions
- If no explicit power analysis was used, you should describe how you decided what sample (replicate) size (number) to use

Sample sizes are described in the material and methods section or figure legends. For animal studies, the number of mice or zebrafish per group was limited to 5-6 to minimize the number of animals used yet provide adequate statistical power. For patient studies, sample size was dependent on availability of our own cohort.

Please outline where this information can be found within the submission (e.g., sections or figure legends), or explain why this information doesn’t apply to your submission:

**Replicates**

- You should report how often each experiment was performed
- You should include a definition of biological versus technical replication
- The data obtained should be provided and sufficient information should be provided to indicate the number of independent biological and/or technical replicates
- If you encountered any outliers, you should describe how these were handled
- Criteria for exclusion/inclusion of data should be clearly stated
- High-throughput sequence data should be uploaded before submission, with a private link for reviewers provided (these are available from both GEO and ArrayExpress)

Minimum number of technical and independent biological replicates was 3.

Please outline where this information can be found within the submission (e.g., sections or figure legends), or explain why this information doesn’t apply to your submission:

**Statistical reporting**

- Statistical analysis methods should be described and justified
- Raw data should be presented in figures whenever informative to do so (typically when N per group is less than 10)
- For each experiment, you should identify the statistical tests used, exact values of N, definitions of center, methods of multiple test correction, and dispersion and precision measures (e.g., mean, median, SD, SEM, confidence intervals; and, for the major substantive results, a measure of effect size (e.g., Pearson's r, Cohen's d)
- Report exact p-values wherever possible alongside the summary statistics and 95% confidence intervals. These should be reported for all key questions and not only when the p-value is less than 0.05.

Statistical analysis methods are described in the material and method section.

Data including error bars are presented as mean ± SD in triplicates unless otherwise stated. Statistical calculations were performed using GraphPad Prism 8.0. Two experimental groups were compared by using unpaired Student’s t tests. Where more than two groups were compared, a one-way ANOVA with Bonferroni’s correction was used. *P* values < 0.05 were considered statistically significant.

Please outline where this information can be found within the submission (e.g., sections or figure legends), or explain why this information doesn’t apply to your submission:

(For large datasets, or papers with a very large number of statistical tests, you may upload a single table file with tests, Ns, etc., with reference to sections in the manuscript.)

**Group allocation**

- Indicate how samples were allocated into experimental groups (in the case of clinical studies, please specify allocation to treatment method); if randomization was used, please also state if restricted randomization was applied
- Indicate if masking was used during group allocation, data collection and/or data analysis

N/A. No clinical trial data were included in this publication.

Please outline where this information can be found within the submission (e.g., sections or figure legends), or explain why this information doesn’t apply to your submission:

**Additional data files (“source data”)**

- We encourage you to upload relevant additional data files, such as numerical data that are represented as a graph in a figure, or as a summary table
- Where provided, these should be in the most useful format, and they can be uploaded as “Source data” files linked to a main figure or table
- Include model definition files including the full list of parameters used
- Include code used for data analysis (e.g., R, MatLab)
- Avoid stating that data files are “available upon request”

Please indicate the figures or tables for which source data files have been provided:

Additional data files are uploaded as supplementary file 1.

Supplementary file 1a. Selected differentially expressed cellular adhesion and ECM-related proteins in omentum metastasis (T) vs normal omentum (O).

Supplementary file 1b: 69 significantly downregulated phosphoproteins shared among three EOC cell lines.

Supplementary file 1c. Selected significantly down- and up-regulated phosphopeptides in comparison between *ΔITGA2* and WT IGROV1 cells.

Supplementary file 1d. Clinical and pathological information of patient samples used in this study

Supplementary file 1e. Clinical and pathological information of patient samples in tissue microarray (TMA)

Figure 7-source data 1. Analysis of phosphoproteomics in IGROV1, SKOV3, and OVCAR3 WT *versus ΔITGA2* cell lines
